# A SEER-based nomogram accurately predicts prognosis in Ewing’s sarcoma

**DOI:** 10.1038/s41598-021-02134-0

**Published:** 2021-11-22

**Authors:** Haibo Zhan, Fengbo Mo, Meisong Zhu, Xiaoyu Xu, Bin Zhang, Hucheng Liu, Min Dai

**Affiliations:** 1 Department of Orthopedics, The First Affiliated Hospital of Nanchang University, Nanchang, 330006 Jiangxi China; 2 Artificial Joints Engineering and Technology Research Center of Jiangxi Province, Nanchang, 330006 Jiangxi China

**Keywords:** Cancer, Computational biology and bioinformatics, Oncology

## Abstract

Ewing's sarcoma is a high-grade malignancy bone and soft tissue tumor that most commonly occurs in children and adolescents. Although the overall prognosis of Ewing's sarcoma has improved, the 5-year survival rate has not improved significantly. The study aimed to determine the risk factors independently associated with the prognosis of Ewing's sarcoma and to construct a nomogram to predict patient survival. Patients diagnosed with Ewing's sarcoma were collected from the Surveillance, Epidemiology, and End Results program database between 2004 and 2015 and further divided into training and validation cohort. Univariate and multivariate Cox regression analyses were used to identify meaningful independent prognostic factors. The nomogram was used to predict 3- and 5-year overall survival (OS) and cancer-specific survival (CSS). Finally, the nomogram was verified internally and externally through the training and validation cohorts, and the predictive capability was evaluated using the receiver operating characteristic (ROC) curve, C-index, and calibration curve and compared with that of the 7th TNM stage. A total of 1120 patients were divided into training (n = 713) and validation (n = 407) cohorts. Based on the multivariate analysis of the training cohort, a nomogram that integrated age, tumor size, primary site, N stage, and M stage was constructed (*P* < 0.05). The predicted C-indexes of OS and CSS of the training cohort were 0.744 (95% CI 0.717–0.771) and 0.743 (95% CI 0.715–0.770), respectively. However, the TNM stage had a C-index of 0.695 (95% CI 0.666–0.724) and 0.698 (95% CI 0.669–0.727) for predicting OS and CSS, respectively. The nomogram showed higher C-indexes than those in the TNM stage. Furthermore, the internal and external calibration curves showed good consistency between the predicted and observed values. Age, tumor size, primary site, N stage, and M stage are independent risk factors affecting the OS and CSS in Ewing’s sarcoma patients. Compared with the 7th TNM staging, the nomogram consisting of these factors was more accurate for risk assessment and survival prediction in patients with Ewing’s sarcoma, thus providing a novel reliable tool for risk assessment and survival prediction in Ewing’s sarcoma patients.

## Introduction

Ewing's sarcoma (ES) is a small round cell sarcoma originating from mesenchymal stem cells, which constitutes 10% to 15% of all bone sarcomas^1^. First proposed by James Ewing in 1921, it is the second most common primary bone and soft tissue malignancy in children and adolescents^2^. It commonly occurs in the backbone of the long bones of the limbs and has a predilection for the femur, tibia, and humerus. It is highly malignant and metastatic and recurs rapidly, thus its extremely poor prognosis^3,4^. Advances in treatment such as in surgery, radiotherapy, and multi-drug chemotherapy have improved the 5-year overall survival (OS) rate of patients with localized ES from approximately 10% to nearly 75%^5^. However, approximately 20–25% of ES patients have metastases at the time of initial diagnosis, and these patients are often resistant to intensive treatment^6^. In addition, the 5-year OS of metastasis patients is only 20–45%, depending on the location of metastasis^4,7^.

ES survival is influenced by different factors, including patient age, primary tumor site, tumor size, distant metastasis, surgery, radiotherapy, and other clinically related prognostic factors^8–12^. According to previous studies, adult age, pelvic involvement, and larger tumor size were associated with poor survival in patients with ES^8,9^. In addition, the occurrence of lung metastasis and extrapulmonary metastasis also significantly increases the risk of poor prognosis for patients^9,10^. Surgery alone is always the best local control method to improve the overall survival rate of patients^11^. Studies have also shown that radiation therapy can only increase the overall survival rate of adults who have not undergone surgery. In patients undergoing surgery, radiation therapy is not associated with a higher overall survival rate for children or adults^12^. However, although previous studies have identified independent prognostic factors of survival in ES, no one independent factor can accurately predict survival of ES patients. Therefore, there is an urgent need to establish a prognostic prediction model that accurately predicts ES survival. However, ES is relatively rare, and thus, it is extremely difficult to conduct large-scale studies.

The Surveillance, Epidemiology, and End Results (SEER) program is a large population-based database for cancer-related epidemiology and health-related service research. It provides data from 18 geographically variable population-based cancer registries, which cover almost 30% of the population of the United States^13^. Nomograms, as simple and reliable predictive tools, have been widely used to assess the prognosis of many cancers. A nomogram integrates various important factors and converts the statistical prediction model into a single numerical estimate of the probability of an event in the form of a chart. These events include the survival rate of a certain disease or the probability of death^14^. Therefore, nomographs have become a reliable tool to guide decision-making and predict the clinical outcomes of many cancers.

This study aimed to determine the risk factors independently associated with ES prognosis and to construct a nomogram to predict patient survival. Towards this goal, we evaluated ES patients registered in the SEER database from 2004 to 2015 and constructed a nomogram based on the clinicopathological data of these patients.

## Materials and methods

### Data source and patients

All patient data were extracted from the US SEER database using SEER*Stat software (version 8.3.5; National Cancer Institute, USA). The SEER database covers approximately 30% of the U.S. population^13^. A total of 3161 ES patients were registered in the database from 2004 to 2015. Among them, we included those who were (1) diagnosed with Ewing sarcoma according to the ICD-O-3/WHO 2008 morphology codes (9260), (2) first diagnosed between 2004 and 2015, (3) with sufficient survival information, and (4) with available follow-up data. The exclusion criteria were as follows: (1) unclear cause of death; (2) unclear metastasis information; (3) unknown race, tumor size, and American joint Committee on Cancer (AJCC) tumor-node-metastasis (TNM) stage; and (4) multiple primary cancers. In total, 1120 ES patients were included in this study. They were randomly according to their year of diagnosis into the training cohort (n = 713, 2004–2011) and the validation cohort (n = 407, 2012–2015). The patient selection flowchart is shown in Supplementary Fig. S1 online.

### Study variables

Data, including the year of diagnosis, age, race, gender, tumor location, tumor size, T stage, N stage, M stage, survival time, cause of death, and survival status, were collected. Given that juveniles and patients aged ≤ 10 years at the time of diagnosis have been reported to have a lower risk of death^15^, the patients were categorized by age at diagnosis into three groups: 0–17 years, 18–59 years, and ≥ 60 years. Race was classified as black, white, and others (American Indian/AK Native, and Asian/Pacific Islander). Tumor-related factors, including tumor location and size, were also investigated. However, data on the original position of the tumor were unclear, and thus we could not confirm the exact position in the bone. As such, we classified the original parts as extremity bones (long and short bones of the extremities), axial bones or skull (spine, pelvis, ribs, mandible, and skull), or others (anterior mediastinum, posterior mediastinum, abdomen, peritoneum, and other soft tissues) based on previous studies^16–18^.

Tumor size was considered as a continuous variable and was classified into the following three categories based on previous studies^18–20^: ≤ 5 cm, 6–10 cm, and > 10 cm. In addition, the T stage was divided into T0, T1, T2, T3, and Tx. The N stage was described as N0 (No), N1 (Yes), and Nx. The M stage was defined as M0 for no metastasis and M1 for positive metastasis.

The study endpoints were the 3- and 5-year rates of overall survival (OS) and cancer-specific survival (CSS).

### Statistical analysis

Categorical data were expressed as frequency and percentage. The chi-square test was used to evaluate the relationship between the demographic and clinical characteristics of the two groups. The chi-square test was performed using SPSS version 22.0 (IBM Corp., Armonk, NY, USA). Survival curves were generated using the Kaplan–Meier method and stratified according to the clinicopathological index. Univariate and multivariate Cox regression analyses were used to identify all risk factors independently associated with OS and CSS. Based on the results of the multivariate Cox regression analysis, a nomogram that integrates all independent factors was constructed to predict the 3- and 5-year OS and CSS. To construct the nomogram, we first used the “coxph” function in the “survival” package to perform univariate and multivariate Cox regression analysis. The 95% confidence interval (CI) and risk ratio were then simultaneously calculated. Significant variables in the multivariate analysis were selected, and we used the “plot” function and the “nom” function in the “rms” package to construct the nomogram model. To interpret the nomogram, a straight line was drawn down to each time point, and then the assigned scores in the range of 0–100 at the top were read. By adding the scores of each selected variable, the probability of individual patient survival can be easily calculated. The C-index was used to assess the prognostic value of the nomogram. The survival curve, receiver operating characteristic (ROC) curve, Harrell’s concordance index (C-index), and calibration curve were determined using the “rms,” “foreign,” and “survival” packages in the R software. The agreement between the actual and predicted nomograms was presented as validation curves. All software packages used in our manuscript were obtained from the website. *P* values < 0.05 were considered statistically significant.

### Ethics approval and consent to participate

The studies involving human participants were reviewed and approved by the medical ethics committee of The First Affiliated Hospital of Nanchang University. Written informed consent from the participants’ legal guardian/next of kin was not required to participate in this study in accordance with the national legislation and the institutional requirements.

## Results

### Patient characteristics and survival

Among the 1120 patients, 40 (3.57%), 989 (88.3%), and 91 (8.13%) patients were black, white, and of other races (American Indian/Alaska Native and Asian/Pacific Islander), respectively. There were 692 (61.79%) males and 428 (38.21%) female patients. There was no significant difference in patient characteristics between the two groups (*P* > 0.05). Table 1 summarizes the baseline clinicodemographic patient characteristics.

**Table 1:** Patients’ demographics, clinical characteristics at diagnosis.

Variables	Training cohort N (%)	Validaton cohort N (%)	Total N(%)	*P*-Value
**N**	713	407	1120	
**Age**				0.288
0–17	364 (51.05)	214 (52.58)	578 (51.61)	
18–59	337 (47.27)	181 (44.47)	518 (46.25)	
≥ 60	12 (1.68)	12 (2.95)	24 (2.14)	
**Race**				0.487
Black	28 (3.93)	12 (2.95)	40 (3.57)	
White	631 (88.50)	358 (87.96)	989 (88.30)	
Other	54 (7.57)	37 (9.09)	91 (8.13)	
**Gender**				0.657
Female	269 (37.73)	159 (39.07)	428 (38.21)	
Male	444 (62.27)	248 (60.93)	692 (61.79)	
**Primary site**				0.578
Axial	341 (47.83)	181 (44.47)	522 (46.61)	
Extremity	327 (45.86)	199 (48.89)	526 (46.96)	
Other	45 (6.31)	27 (6.63)	72 (6.43)	
**Tumor size**				0.480
≤ 5 cm	182 (25.53)	107 (26.29)	289 (25.80)	
6–10 cm	298 (41.80)	181 (44.47)	479 (42.78)	
> 10 cm	233 (32.68)	119 (29.24)	352 (31.43)	
**Metastasis**				0.869
No	510 (71.53)	293 (72.00)	803 (71.70)	
Yes	203 (28.47)	114 (28.00)	317 (28.30)	
**T stage**				0.363
T0	3 (0.42)	4 (0.98)	7 (0.63)	
T1	302 (42.36)	182 (44.72)	484 (43.21)	
T2	372 (52.17)	202 (49.63)	574 (51.25)	
T3	17 (2.38)	13 (3.19)	30 (2.68)	
Tx	19 (2.66)	6 (1.47)	25 (2.23)	
**N stage**				0.080
N0	619 (86.82)	361 (88.70)	980 (87.50)	
N1	50 (7.01)	35 (8.60)	85 (7.59)	
Nx	44 (6.17)	11 (2.70)	55 (4.91)	

**Figure 1:**
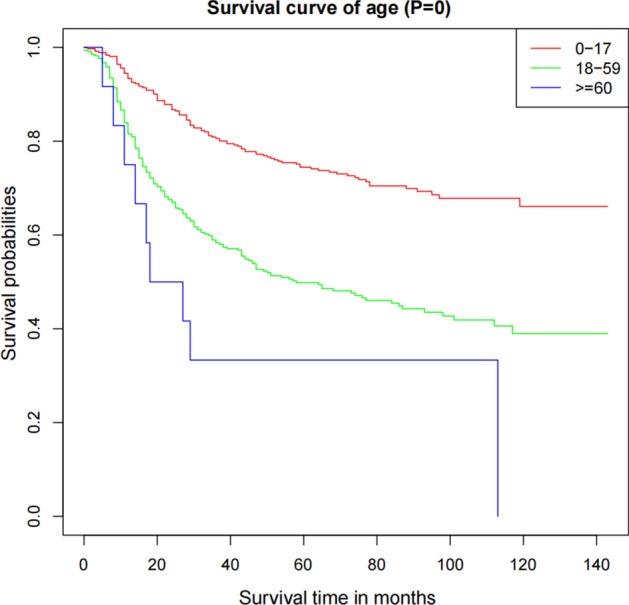
Kaplan–Meier curve for the training cohort patients according to the age.

The Kaplan–Meier survival curve showed that patients aged 0–17 years have a better prognosis than that in patients aged 18–59 years and ≥ 60 years (*P* < 0.001) (Fig. 1). Race, gender, primary site, tumor size, T stage, N stage, and M stage were identified as prognostic factors (see Supplementary Figs. S2 and S3 online).

### Prognostic nomograms for OS and CSS

Univariate Cox regression analysis of OS showed that age, gender, primary site, tumor size, N stage, and M stage were significant influencing factors (*P* < 0.05) (Tables 2 and 3). Meanwhile, only age, primary site, tumor size, N stage, and M stage were significant predictors of CSS (*P* < 0.05). In multivariate Cox regression analysis, the independent prognostic factors of OS and CSS were similar; these were age, primary site, tumor size, N stage, and M stage (*P* < 0.05). For instance, compared with patients aged < 17 years, those aged > 60 years had a significantly higher risk of death (HR: 6.583, 95% CI 3.293–13.157).

**Table 2:** Univariate analysis and Multivariate analysis of variables for OS in patients.

Variables	Univariate Analysis HR (95%CI)	*P*-Value	Multivariate Analysis HR (95%CI)	*P*-Value
**Age**				
0–17	Reference	–	Reference	–
18–59	2.368 (1.861–3.012)	0.000*	2.215 (1.732–2.833)	0.000*
≥ 60	4.229 (2.139–8.361)	0.000*	6.583 (3.293–13.157)	0.000*
**Race**				
Black	Reference	–	–	–
White	0.640 (0.380–1.077)	0.093	–	–
Other	0.848 (0.451–1.594)	0.608	–	–
**Gender**				
Female	Reference	–	Reference	–
Male	1.285 (1.008–1.639)	0.043*	1.045 (0.815–1.339)	0.730
**Primary site**				
Axial	Reference	–	Reference	–
Extremity	0.649 (0.508–0.828)	0.000*	0.685 (0.535–0.877)	0.003*
Other	1.253 (0.823–1.910)	0.294	0.942 (0.609–1.458)	0.789
**Tumor size**				
≤ 5 cm	Reference	–	Reference	–
6–10 cm	1.559 (1.119–2.171)	0.009*	1.341 (0.959–1.876)	0.086
> 10 cm	2.686 (1.935–3.730)	0.000*	1.939 (1.378–2.729)	0.000*
**Metastasis**				
No	Reference	–	Reference	–
Yes	3.953 (3.134–4.985)	0.000*	3.149 (2.458–4.035)	0.000*
**T stage**				
T0	Reference	–	–	–
T1	0.289 (0.040–2.082)	0.218	–	–
T2	0.613 (0.086–4.385)	0.626	–	–
T3	1.245 (0.163–9.529)	0.833	–	–
Tx	0.737 (0.093–5.830)	0.772	–	–
**N stage**				
N0	Reference	–	Reference	–
N1	2.017 (1.386–2.933)	0.000*	1.483 (1.007–2.184)	0.046*
Nx	1.799 (1.191–2.717)	0.005*	1.116 (0.728–1.710)	0.616

HR, Hazard Ratio; CI, Confidence Interval. *means *p* < 0.05.

**Table 3:** Univariate analysis and Multivariate analysis of variables for CSS in patients.

Variables	Univariate analysis HR (95%CI)	*P*-value	Multivariate Analysis HR (95%CI)	*P*-Value
**Age**				
0–17	Reference	–	Reference	–
18–59	2.382 (1.862–3.046)	0.000*	2.245 (1.746–2.886)	0.000*
≥ 60	3.934 (1.913–8.090)	0.000*	6.130 (2.950–12.737)	0.000*
**Race**				
Black	Reference	–	–	–
White	0.657 (0.383–1.127)	0.127	–	–
Other	0.877 (0.458–1.680)	0.692	–	–
**Gender**				
Female	Reference	–	–	–
Male	1.278 (0.997–1.638)	0.053	–	–
**Primary site**				
Axial	Reference	–	Reference	–
Extremity	0.659 (0.514–0.845)	0.001*	0.698 (0.543–0.898)	0.005*
Other	1.151 (0.737–1.799)	0.536	0.866 (0.546–1.374)	0.541
**Tumor size**				
≤ 5 cm	Reference	–	Reference	–
6–10 cm	1.600 (1.138–2.249)	0.007*	1.371 (0.972–1.934)	0.073
> 10 cm	2.715 (1.937–3.807)	0.000*	1.957 (1.379–2.776)	0.000*
**Metastasis**				
No	Reference	–	Reference	–
Yes	3.996 (3.153–5.066)	0.000*	3.205 (2.490–4.125)	0.000*
**T stage**				
T0	Reference	–	–	–
T1	0.281 (0.039–2.025)	0.208	–	–
T2	0.592 (0.083–4.238)	0.602	–	–
T3	1.259 (0.164–9.630)	0.825	–	–
Tx	0.748 (0.095–5.920)	0.784	–	–
**N stage**				
N0	Reference	–	Reference	–
N1	2.033 (1.389–2.976)	0.000*	1.515 (1.023–2.243)	0.038*
Nx	1.722 (1.121–2.644)	0.013*	1.081 (0.695–1.683)	0.729

HR, Hazard Ratio; CI, Confidence Interval. *means p < 0.05.

### Construction and Validation of the Nomogram

**Figure 2:**
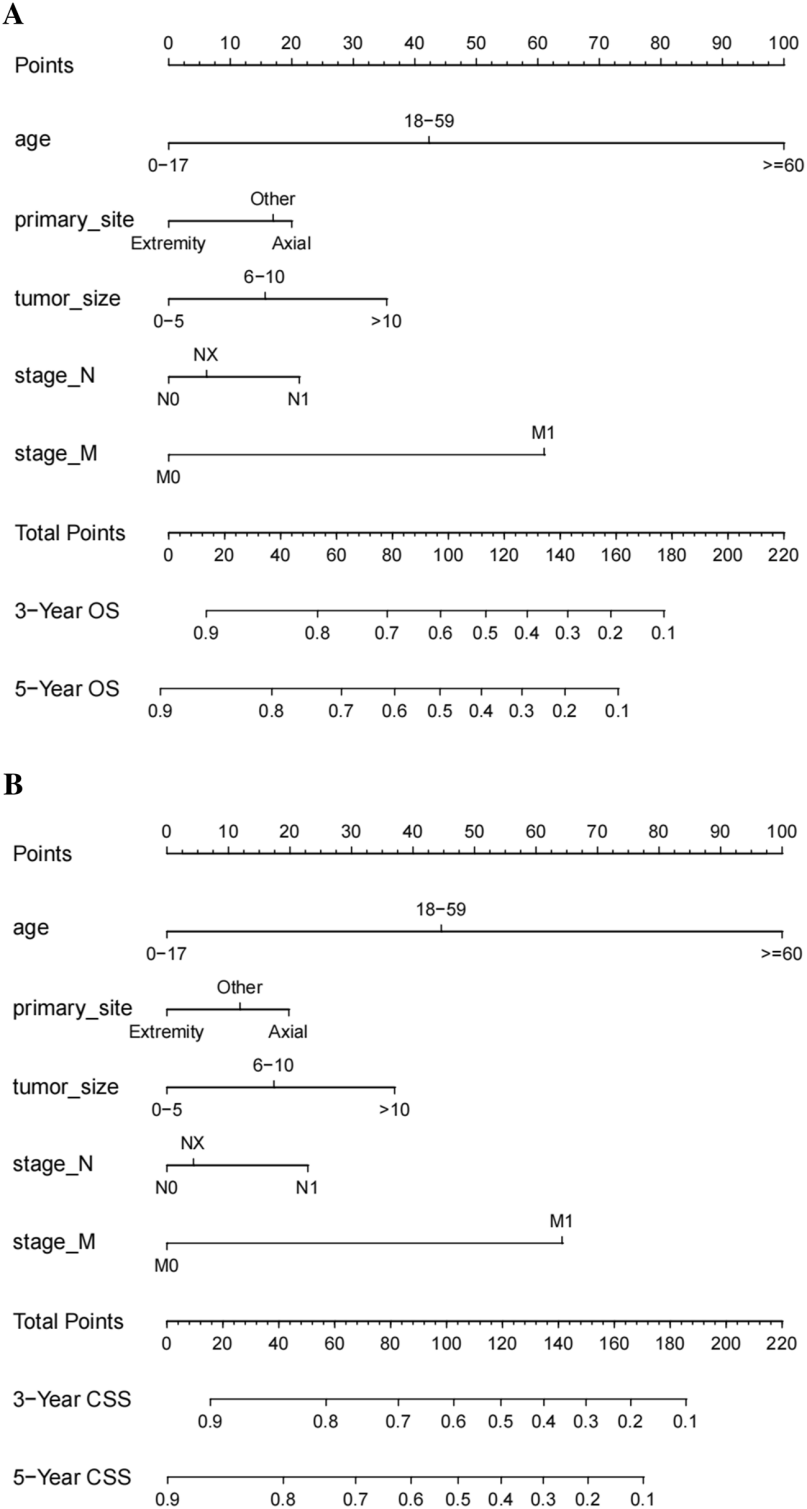
Nomograms for predicting the 3- and 5-year overall survival (**A**) and cancer-specific survival (**B**) of ES patients.

Age, primary tumor site, tumor size, N stage, and M stage were used as prognostic predictors to construct the nomogram (Fig. 2). The strongest influencing factor of prognosis was age, followed by M stage and tumor size, and the primary site had the least influence. In the internal validation, the nomogram had a C-index of 0.744 (95% CI 0.717–0.771) and 0.743 (95% CI 0.715–0.770) for predicting OS and CSS, respectively. The corresponding values in the external validation were 0.803 (95% CI 0.748–0.858) and 0.804 (95% CI 0.747–0.861), respectively (Table 4). Moreover, the nomogram showed higher C-indexes than those in the TNM stage in both external and internal validations. In the internal validation, the TNM stage had a C-index of 0.695 (95% CI 0.666–0.724) and 0.698 (95% CI 0.669–0.727) for predicting OS and CSS, respectively. The corresponding values in the external validation were 0.714 (95% CI 0.636–0.792) and 0.732 (95% CI 0.656–0.808), respectively (Table 4). It was suggested that the nomogram was more effective than TNM stage in predicting the survival of patients.

**Table 4:** Accuracy of the prediction score of the nomogram and TNM stage for estimating prognosis of patients.

Variables	Training set		Validation set	
	OS (95%CI)	CSS (95%CI)	OS (95%CI)	CSS (95%CI)
C index for nomogram	0.744 (0.717–0.771)	0.743 (0.715–0.770)	0.803 (0.748–0.858)	0.804 (0.747–0.861)
C index for TNM stage	0.695 (0.666–0.724)	0.698 (0.669–0.727)	0.714 (0.636–0.792)	0.732 (0.656–0.808)

OS, Overall Survival; CSS, Cancer-specific Survival; CI, Confidence Interval.

**Figure 3:**
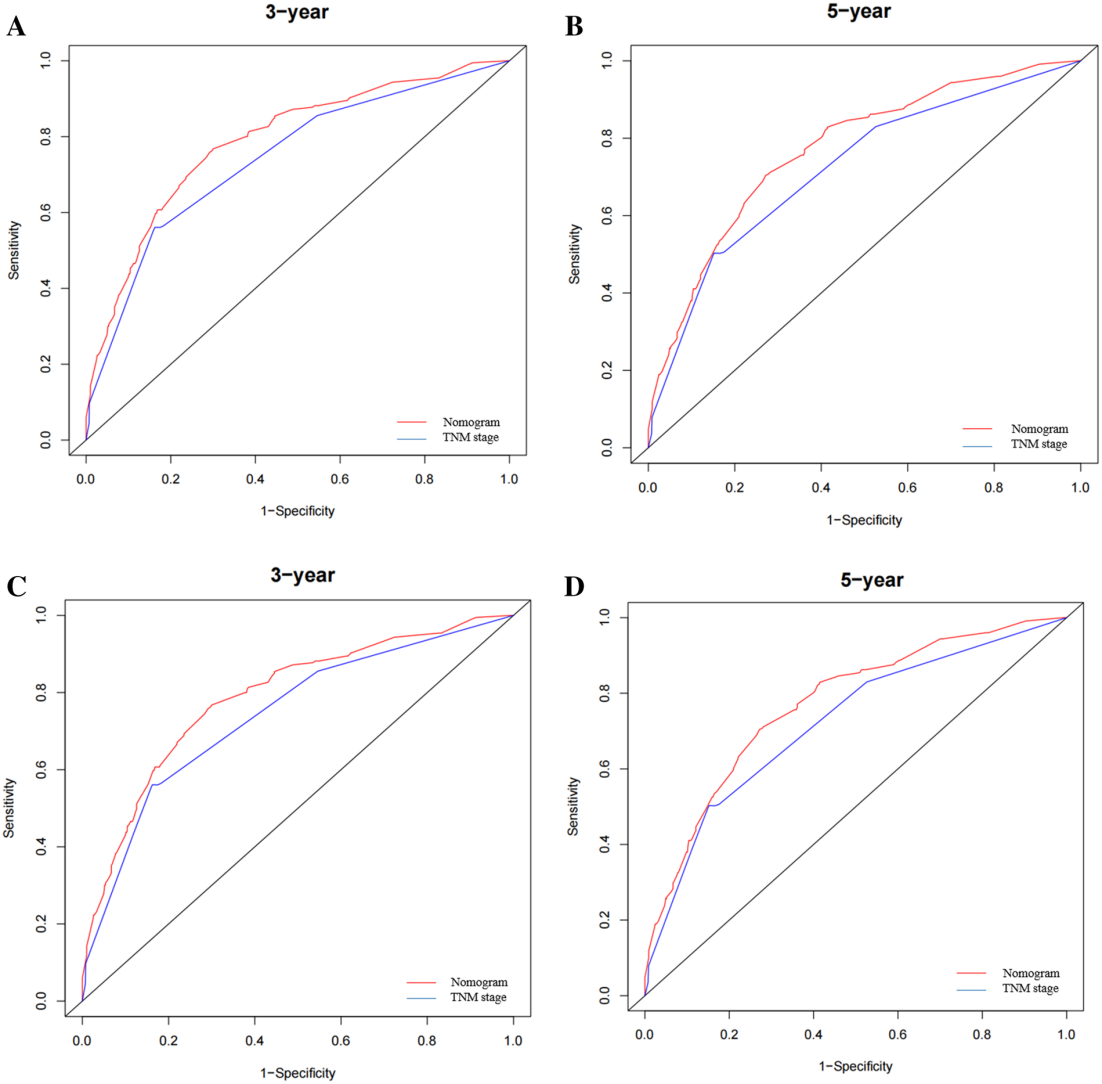
ROC curve of the nomogram and 7th TNM stage in predicting the prognosis of training cohort and validation cohort patients. (**A**, **B**) ROC curve for the 3- and 5-year points in the training cohort. (**C**, **D**) ROC curve for the 3- and 5-year points in the validation cohort.

**Figure 4:**
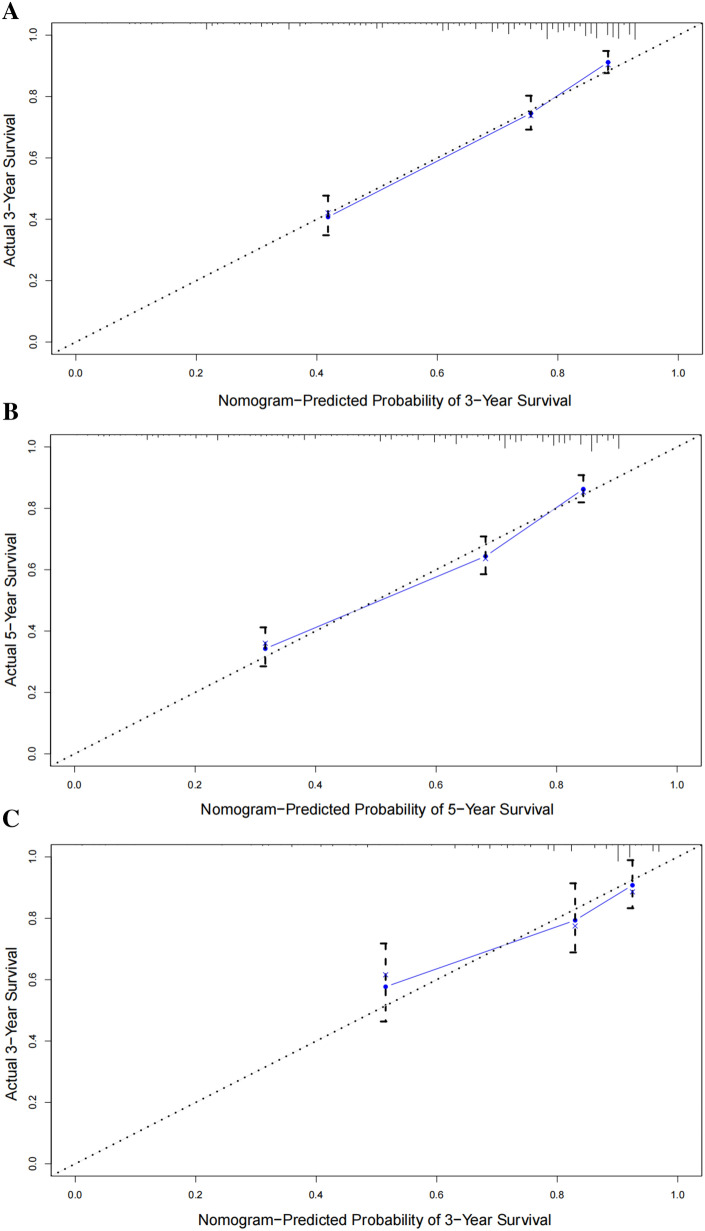
The calibration curve for predicting patient overall survival at 3-year (**A**) and 5-year (**B**) in the training cohort and at 3-year (**C**) in the validation cohort. The 45-degree line means an ideal match between the actual survival (y-axis) and the predicted survival of the nomogram (x-axis). The vertical line represents 95% confidence intervals.

The area under the curves (AUCs) of the nomogram in predicting the 3- and 5-year OS were 0.788 and 0.771, respectively, while those of the TNM stage were 0.742 and 0.722, respectively. In the external validation, the AUCs of the nomogram for predicting the 3- and 5-year OS were 0.734 and 0.756, respectively, while those of the TNM stage were 0.663 and 0.654, respectively. These results indicate that the nomogram can better predict the 3- and 5-year prognoses of ES patients (Fig. 3). Furthermore, the internal and external calibration curves showed a significant agreement between the predicted and observed values (Fig. 4).

## Discussion

ES is a rare and aggressive malignant tumor that is the second most common primary bone and soft tissue malignancy in children and adolescents, with the incidence second only to osteosarcoma^3,4^. ES has a poor prognosis, with a survival rate of 70%–80% for local disease and only 30% for metastatic disease^5,6^. Although treatment advances have improved ES survival, accurate prognostic prediction remains challenging. Furthermore, although it is the second most common malignancy in children and young adults, ES is extremely rare, occurring in only 2 per 1 million children and adolescents worldwide. Thus, large-scale research is challenging. In this study, we used a large-sample database of the SEER program and established a predictive nomogram for the prognosis of ES patients. The nomogram integrated routinely available information such as age, primary site, tumor size, N stage, and M stage to predict OS and CSS in a large cohort of 1120 patients with ES.

Consistent with previous reports^8,9,11^, age was identified as an independent risk factor for poor prognosis in this study. Patients over 60 years had lower OS and CSS than those of patients aged 18–59 years and < 17 years; the younger the age, the better the prognosis. This could be because older adult patients are more likely to develop metastatic disease and receive lower doses of chemotherapy because of their low tolerance to chemotherapy^8,21^. Moreover, older adult patients may have multiple comorbidities, including diabetes, hypertension, and heart disease, worsening their prognosis. Tumor size and axial primary tumors were also identified as independent risk factors of prognosis. Multivariate analysis showed that OS and CSS were significantly lower in patients with larger tumors (> 10 cm) and axial primary tumors, consistent with previous studies. For instance, Duchman et al.^18^ found that ES patients with metastatic disease, axial tumor site, and tumor diameter > 10 cm had a significantly lower 10-year CSS. Axial primary tumors and larger tumors are also often associated with metastatic disease^22^, and metastatic disease has been established to have a direct impact on OS and CSS^23^.

ES is an aggressive tumor with a high rate of local recurrence and distant metastasis, and these metastases are often resistant to intensive treatment. Moreover, axial primary tumors are usually closer to large blood vessels, increasing the possibility of distant metastatic disease. However, patients with axial primary tumors usually do not have obvious symptoms, thus delaying diagnosis and increasing the risk of distant metastasis^17,18^. The current study found that gender, race, and T stage were not risk factors related to patient prognosis. Meanwhile, patients aged > 60 years and with larger tumors (> 10 cm), axial primary tumors, advanced N stage, and metastatic disease at the time of diagnosis are more likely to have poor OS and CSS.

Nomograms have been shown to predict the survival of many tumor types and are considered to be more accurate than the 7th AJCC staging system^14^. The nomogram in this study included age, tumor size, primary site, N stage, and M stage. Its predictive accuracy for the 3- and 5-year OS and CSS was evaluated by comparing the predicted survival and actual survival rate. In the training cohort, the C-indices for OS and CSS were 0.744 and 0.743, respectively. In the verification group, the corresponding values were 0.803 and 0.804, respectively, indicating the reliability of the nomogram. The ROC curves also indicated that our nomogram better predicts the OS and CSS than the 7th TNM staging system. The internal and external calibration curves showed a significant agreement between the predicted and observed values. Collectively, these findings indicate that the nomogram can be helpful to evaluate patient prognosis and determine the need for further chemotherapy after surgery to improve patient outcomes.

Our study has some limitations. First, the TNM stage was according to the 7th AJCC staging system, which is not up-to-date and may reduce effectiveness. Second, our nomogram is only constructed based on data from the SEER database where some patient data will inevitably be lost. This may reduce the number of qualified cases and may lead to the risk of selection bias. Finally, because there is no information on blood biomarkers such as serum lactate dehydrogenase (LDH), alkaline phosphatase, and carcinoembryonic antigen (CEA) in the SEER database, and thus these were not included in the analysis. Some previous studies have shown that combining blood biomarkers, such as hemoglobin, neutrophils, and LDH, can improve the predictive capability of the nomogram^24^. We will include these data in future research to improve the nomogram. Despite the limitations, the data in this study can be used as reference to develop a globally applicable predictive model of ES prognosis.

## Conclusions

Age, tumor size, primary site, N stage, and M stage are independent risk factors affecting the OS and CSS in ES patients. Compared with the 7th TNM staging, the nomogram consisting of these factors was more accurate for risk assessment and survival prediction in patients with ES, thus providing a novel reliable tool for risk assessment and survival prediction in ES patients.

## Supplementary Information

Supplementary Information.

## Data Availability

Publicly available datasets were analyzed in this study. These data can be found here: https://seer.cancer.gov/. The datasets supporting the conclusions of this article are included within the article.

## Abbreviations

AUC: Area under the curve
CEA: Carcinoembryonic antigen
CI: Confidence interval
CSS: Cancer-special survival
ES: Ewing’s sarcoma
OS: Overall survival
ROC: Receiver operating characteristic
SEER: Surveillance, epidemiology, and end results
