# Supplementary material for: A SEER-based nomogram accurately predicts prognosis in Ewing’s sarcoma
Source: Sci Rep. 2021 Nov 22;11:22723. doi: 10.1038/s41598-021-02134-0 (PMC8608824; doi:10.1038/s41598-021-02134-0)

A SEER-based nomogram accurately predicts prognosis in Ewing sarcoma

Haibo Zhan^1,2#^, Fengbo Mo^1,2#^, Meisong Zhu^1,2^, Xiaoyu Xu^1,2^, Bin Zhang^1,2^, Hucheng Liu^1,2*^ and Min Dai^1,2*^

^1^Department of Orthopedics, The First Affiliated Hospital of Nanchang University, Nanchang, Jiangxi 330006, China; ^2^Artificial Joints Engineering and Technology Research Center of Jiangxi Province, Nanchang, Jiangxi 330006, China

Email:

Haibo Zhan: [HBZzhanhaibo@163.com](mailto:HBZzhanhaibo@163.com)

Fengbo Mo: [doctormfb@126.com](mailto:565510953@qq.com)

Meisong Zhu: [zhumeisongv@163.com](mailto:zhumeisongv@163.com)

Xiaoyu Xu: [614036234@qq.com](mailto:614036234@qq.com)

Bin Zhang: Drbinzhangg@163.com

**^#^**These authors contributed to the work equally and should be regarded as co-first authors;

^*^Corresponding author: **Hucheng Liu,** Dr., Department of Orthopedics, The First Affiliated Hospital of Nanchang University, Artificial Joints Engineering and Technology Research Center of Jiangxi Province, No.17 Yongwaizheng Street, Nanchang 330006, Jiangxi, China. E-mail [lliuhucheng@163.com](mailto:lliuhucheng@163.com); **Min Dai**, Professor, Dr., Department of Orthopedics, Artificial Joints Engineering and Technology Research Center of Jiangxi Province, The First Affiliated Hospital of Nanchang University, No.17 Yongwaizheng Street, Nanchang, Jiangxi 330006, China; E-mail: [daimin@medmail.com.cn](mailto:daimin@medmail.com.cn).

**Supplementary Fig. S1** The flow chart of extracted patients from the SEER database.


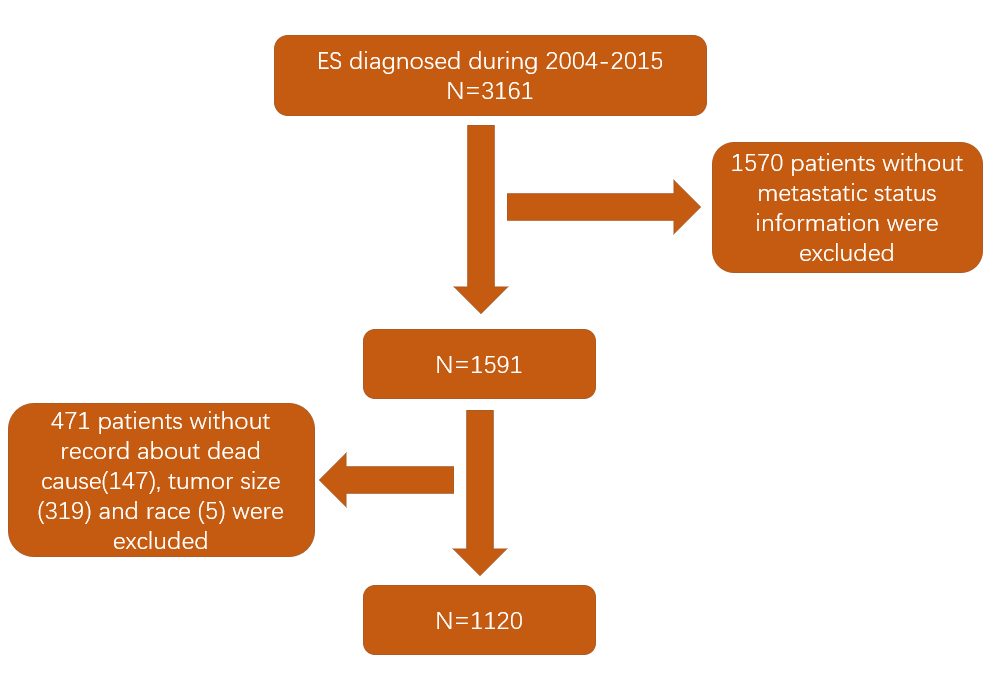


**Supplementary Fig. S2** Kaplan–Meier curve for the training cohort patients according to different variables. (A) Gender, (B) race, (C) primary site, (D) tumor size.


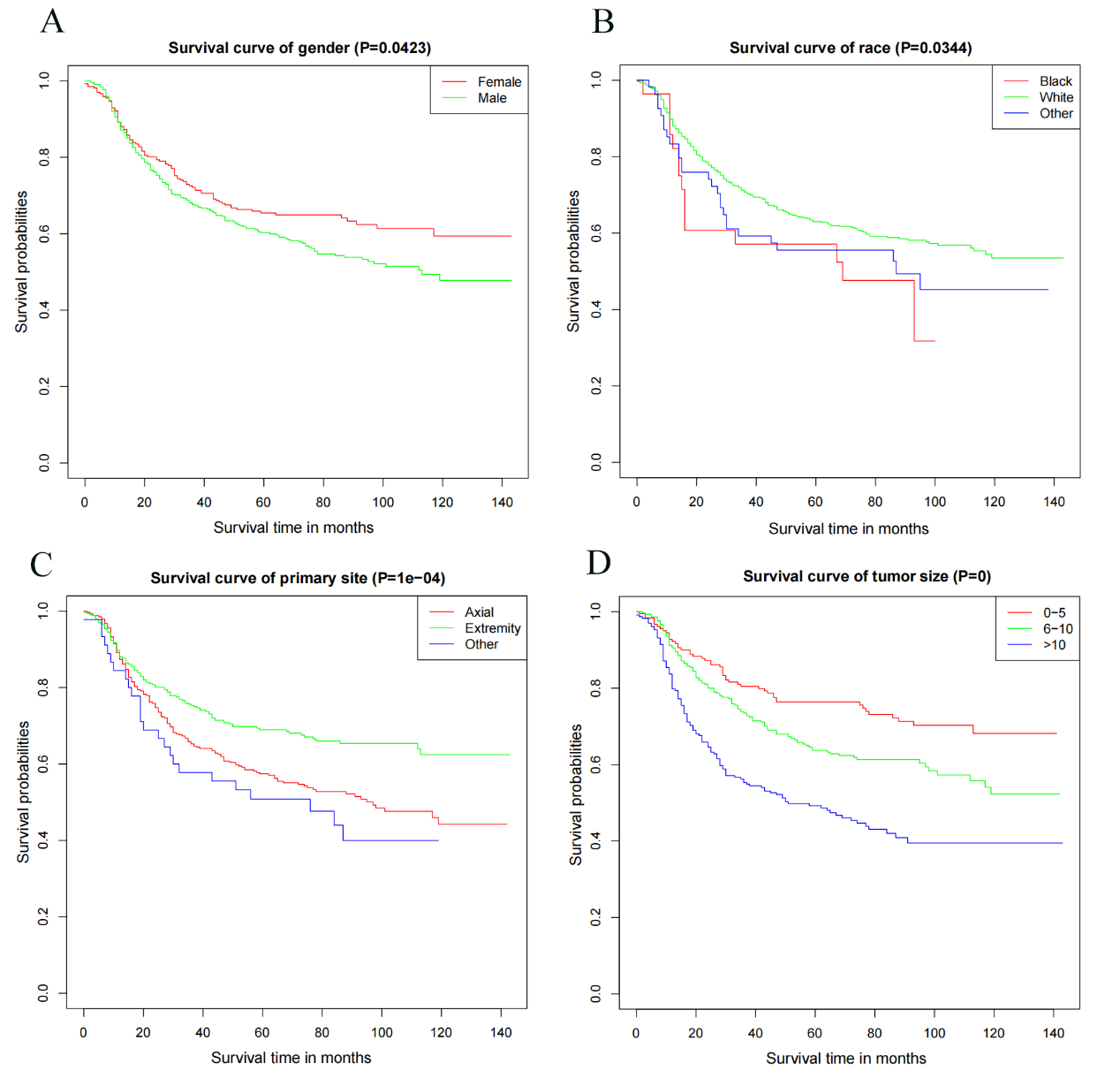


**Supplementary Fig. S3** Kaplan–Meier curve for the training cohort patients according to different variables. (A) T stage, (B) N stage, (C) M stage.


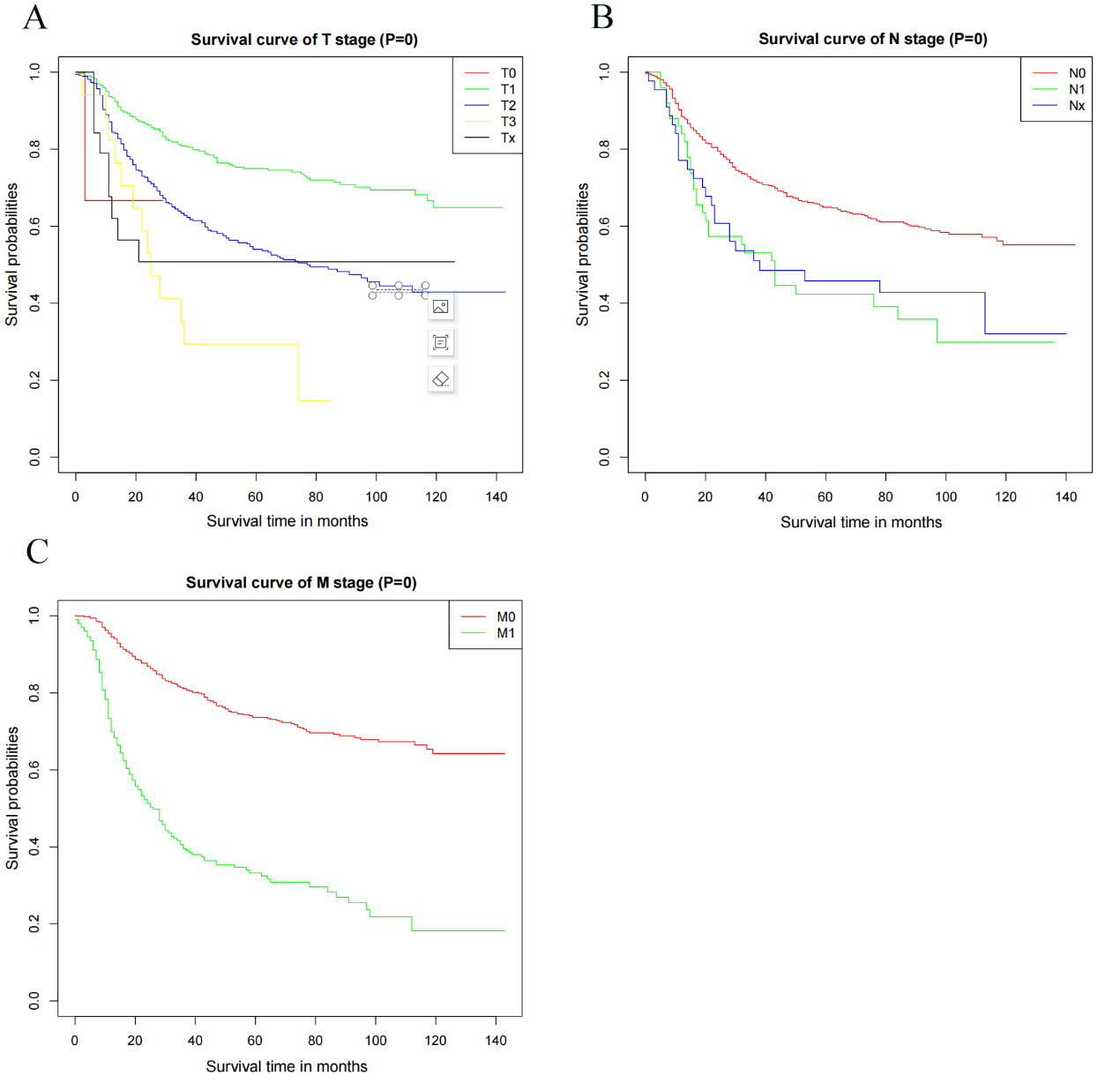

Supplement: Supplementary file 1 — Supplementary Information. [file 41598_2021_2134_MOESM1_ESM.docx]
